# Neurally Derived Tissues in *Xenopus laevis* Embryos Exhibit a Consistent Bioelectrical Left-Right Asymmetry

**DOI:** 10.1155/2012/353491

**Published:** 2012-12-30

**Authors:** Vaibhav P. Pai, Laura N. Vandenberg, Douglas Blackiston, Michael Levin

**Affiliations:** Department of Biology and Tufts Center for Regenerative and Developmental Biology, Tufts University, Medford, MA 02155, USA

## Abstract

Consistent left-right asymmetry in organ morphogenesis is a fascinating aspect of bilaterian development. Although embryonic patterning of asymmetric viscera, heart, and brain is beginning to be understood, less is known about possible subtle asymmetries present in anatomically identical paired structures. We investigated two important developmental events: physiological controls of eye development and specification of neural crest derivatives, in *Xenopus laevis* embryos. We found that the striking hyperpolarization of transmembrane potential (*V*_mem_) demarcating eye induction usually occurs in the right eye field first. This asymmetry is randomized by perturbing visceral left-right patterning, suggesting that eye asymmetry is linked to mechanisms establishing primary laterality. Bilateral misexpression of a depolarizing channel mRNA affects primarily the right eye, revealing an additional functional asymmetry in the control of eye patterning by *V*_mem_. The ATP-sensitive K^+^ channel subunit transcript, SUR1, is asymmetrically expressed in the eye primordia, thus being a good candidate for the observed physiological asymmetries. Such subtle asymmetries are not only seen in the eye: consistent asymmetry was also observed in the migration of differentiated melanocytes on the left and right sides. These data suggest that even anatomically symmetrical structures may possess subtle but consistent laterality and interact with other developmental left-right patterning pathways.

## 1. Introduction

Consistent (directionally biased) left-right asymmetry of viscera, heart, and brain is overlaid upon the overall bilaterally symmetric body plan of a wide range of organisms [1, 2]. Errors in this process form an important class of human birth defects [3–5]. Thus, understanding left-right patterning and the interaction of individual organ systems with the axial polarity of the body is of great interest for both basic evolutionary developmental biology and for the biomedicine of birth defects. Likewise, the highly lateralized functions of the brain (e.g., language, speech, and handedness) and their partial disconnect from anatomical left-right asymmetry have been a fascinating topic under study for several decades [6, 7].

Recent studies have identified numerous genetic [8–13], biophysical [14–16], and physiological [17–21] mechanisms that underlie large-scale left-right asymmetry and the *situs* of asymmetric organs. However, much less well-understood are subtle asymmetries occurring in paired body structures which are anatomically symmetrical [14].

Such asymmetries manifest in several ways. First, quantitative morphometrics of paired structures can reveal cryptic polarity that may not be apparent from gross morphological examination [22]; insect wings are a good example of this phenomenon [23, 24], as are human foot size [25] and sex organ placement [26]. It is crucial to note that such biased examples of left-right patterning (where the asymmetry is coordinated in a consistent fashion with the other two major axes) are a distinct phenomenon from fluctuating asymmetry, which involves simple differences between the left and right sides derived from developmental noise [27, 28].

Second, differences of gene expression have been described in seemingly symmetric body structures. These include consistent differences in the timing of highly dynamic bilateral transcriptional waves that drive the segmentation clock of somitogenesis [29–31], as well as long-term asymmetries in expression of markers such as EGF-like growth factors and MLC3F [32, 33].

Perhaps the most interesting types of subtle asymmetries are those that are revealed only under functional perturbation. It has long been known that consistently sided unilateral limb defects are induced in rodents by some compounds such as cadmium [34–36]. Spontaneous genetic defects sometimes uncover biased asymmetries, as seen in several human syndromes that unilaterally affect the limbs [37], face [38], or hips [39]. Holt-Oram syndrome (Tbx5 related) presents upper limb malformations which are much more common on the left side [37, 40–42], while fibular hypoplasia affects the right side more often [43]. Indeed, a variety of human syndromes affecting paired organs have a significant bias for one side [39]. Curiously, unilateral defects in otherwise symmetrically placed structures (e.g., teeth) in monozygotic twins exhibit a mirroring-opposite sidedness in the two twins (reviewed in [44]). Hemihyperplasia [45, 46], a rare phenomenon where one side of the body abnormally overgrows, is right biased [47]. Importantly, targeted molecular-genetic experiments in tractable model systems are beginning to reveal entry points into this process; for example, attenuated FGF8 signaling results in consistently biased left-right asymmetric development of the pharyngeal arches and craniofacial skeleton in zebrafish [48]. 

Most studies of asymmetry use cardiac and visceral *situs* as readout, focusing on mechanisms that determine the laterality of major body organs. However, the consistent asymmetries revealed in the above examples hint at the possibility that left-right identity (resulting from tissues' interactions with the pathways that establish the left-right axis) could be far more prevalent throughout the body than is currently appreciated. What signaling pathways might underlie laterality information in anatomically symmetrical tissues? While most work on morphogenetic controls focuses on biochemical pathways [49, 50] and physical forces [51, 52], exciting recent as well as classical data demonstrate the importance of endogenous bioelectrical determinants of cell behavior and large-scale patterning [53–59]. We thus focused our search on asymmetries that manifest at the level of functional physiology.

Prior work in the planarian flatworm model system revealed that during head regeneration, the right eye was significantly more sensitive to inhibition of the H, K-ATPase ion pump than the left, in terms of frequency of induced patterning defects [60]. In our efforts to understand the functions of spatial gradients in transmembrane potential (*V*_mem_) during vertebrate development and regeneration [18, 61–65], we examined eye development in embryos of *Xenopus laevis*. Using a noninvasive assay with a fluorescent reporter of *V*_mem_ [66, 67], it was found that the nascent eye fields are demarcated by a localized hyperpolarization of *V*_mem_ [68, 69]; strikingly, this physiological signature of eye fate is consistently biased, with cells on the right side of the midline hyperpolarizing first. We also report similar consistent subtle asymmetries in the migratory behavior of neural crest-derived melanocytes. Here, we molecularly characterize this novel asymmetry, revealing that physiological analysis can uncover cryptic differences in patterning information not apparent from molecular marker analysis or anatomical examination. Understanding such subtle functional asymmetries may become useful in addressing and targeting diseases with asymmetric manifestations.

## 2. Results

### 2.1. Eye Field Cells Exhibit a Consistently Asymmetric *V*_*mem*_

**Figure 1:**
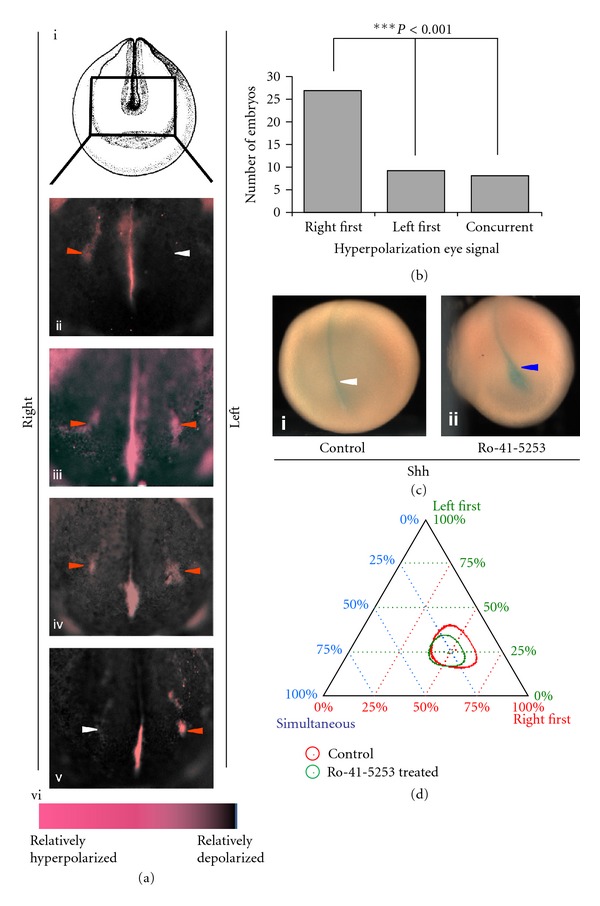
Rightward bias in induction of polarization signal regulating eye development. (a) Incubation in voltage-sensitive CC2-DMPE dye of multiple *Xenopus* embryos tracked from stage 18 to stage 20 shows the representative temporal progression of hyperpolarization signal (red arrowheads) during development (ii)–(v). White arrowheads indicate the lack of a coherent (contiguous) spot of signal. (vi) Color bar representing the scale of relative depolarization and hyperpolarization as seen with the CC2-DMPE dye. (b) Bar graph comparing a group of *Xenopus *embryos (*n* = 44) that were tracked individually and analyzed for the first detectable hyperpolarization signal using CC2-DMPE; a significant bias is observed favoring the right side. A pairwise comparison and Chi-squared test analysis were done between the groups. (c) *In situ* hybridization analysis of Sonic Hedgehog (Shh) signal in stage 18 *Xenopus* embryos either untreated (control) ((i) white arrowhead) or treated with 1.5 *μ*M retinoic acid receptor inhibitor Ro-41-5253 ((ii) blue arrowhead) from midgastrula stage. Ro-41-5253 treatment significantly enhances the Shh expression signal in 92% of treated embryos (*n* = 29). (d) Categorical data analysis using a ternary plot shows that the treatment with Ro-41-5253 (1.5 *μ*M) that inhibits retinoic acid receptor signal resulted in no significant change in the rightward bias of polarization signal (as observed via CC2-DMPE staining) involved in *Xenopus* eye development. In control (untreated) embryos the polarization was 51.5% right first, 26.5% left first, and 22% simultaneous (*n* = 72). In the Ro-41-5253-treated embryos the polarization was 48% right first, 25% left first, and 27% simultaneous (*n* = 126). The circles in the plot represent 95% confidence intervals. Using the calculations provided by a ternary plot algorithm (https://webscript.princeton.edu/~rburdine/stat/three_categories), the results are statistically significant only when there is no overlap of the confidence intervals.

*V*_mem_ plays a crucial functional role in defining the eye fields during the development of the *Xenopus *embryo [68, 70]. Using the fluorescent voltage-sensitive reporter dye CC2-DMPE [71] *in vivo* to characterize real-time changes in membrane potential [17, 65, 72], we discovered a novel physiological asymmetry between the left and right eye primordia. At stage 18, normal *Xenopus* embryos exhibit bilateral clusters of cells with a more strongly polarized *V*_mem_ than their neighbors around the putative eye region (Figure 1(a), red arrowheads [68]). Surprisingly, real-time imaging analysis revealed that the right eye field is polarized first, followed by polarization of the left eye field (Figures 1(a) and 1(b)). Though not absolute, this physiological asymmetry is consistently biased, since more than 3-fold as many embryos polarized first on the right compared to those that began polarization on the left (Figure 1(b); *N* = 44, *P* < 0.001, Chi-squared test comparison with an unbiased expectation). In addition to the significant asymmetry at one key timepoint, we followed 6 individual embryos (imaged through eye development stages) to estimate the temporal difference between hyperpolarization events on the two sides. At 20°C, 4 embryos exhibited a 30 min delay in the hyperpolarization of the left eye spot, while 2 embryos exhibited a 20 min delay in the appearance of the left eye spot hyperpolarization. We conclude that eye development in *Xenopus* is inherently asymmetric, with the right eye field most often initiating the endogenous program of polarization.

Previous studies in chick and mouse embryos have shown that somites, though anatomically symmetrical, also exhibit an underlying asymmetry [30, 73]. Somites are shielded from this inherent asymmetry by the action of retinoic acid (RA) signaling, resulting in symmetrical development. In order to determine whether the asymmetry in eye signals is due to an incomplete shielding effect of RA-dependent signaling, we treated embryos with 1.5 *μ*M of the broad-spectrum RA receptor inhibitor Ro-415253. Ro-41-5253 at this concentration has previously been shown to affect RA signaling in *Xenopus* [74]. We used a previously documented midline marker—Sonic Hedgehog (Shh), to further confirm the effect of Ro-41-5253. *Xenopus* embryos were left untreated (Controls) or treated with Ro-41-5253 (1.5 *μ*M), and the Shh expression was evaluated at stage 18 by *in situ* hybridization (Figure 1(c)). Control embryos showed Shh expression as a thin line along the dorsal midline (Figure 1(c), (i) white arrowhead). However, Ro-41-5253-treated embryos (>90%) showed a clear increase in Shh expression along the midline (Figure 1(c), (ii) blue arrowhead) as previously documented [74]. This change in Shh expression confirms that at 1.5 *μ*M Ro-41-5253 is effective in inhibiting RA receptor signaling.

The effect of Ro-41-5253 on the asymmetric eye polarization signal was analyzed in stage 18 embryos using the CC2-DMPE voltage reporter dye. Each treatment (control and Ro-41-5253) was plotted on a ternary graph using three measures of eye-related polarization signal (right first, left first, and simultaneous) according to the methods described previously [75] (Figure 1(d)). The circles in the plot represent respective error regions of 95% confidence intervals. Using the calculations provided by a ternary plot algorithm (https://webscript.princeton.edu/~rburdine/stat/three_categories), the results are considered statistically significant (*P* < 0.05) when there is no overlap of the 95% confidence intervals. Analysis of the polarization signal in the Ro-41-5253-treated embryos at stage 18 showed a significant right side first bias (*n* = 126) (right first 48%, left first 25%, and simultaneous 27%) (Figure 1(d), green circle), similar to that of control/untreated embryos (*n* = 72) (right first 51.5%, left first 26.5%, and simultaneous 22%) (Figure 1(d), red circle; *P* > 0.05, ternary plot algorithm). Thus, RA signaling does not play a role in the right-side-first bias of *V*_mem_ signal in the eye primordia.

### 2.2. Eye Field *V*_*mem*_ Asymmetry Is Perturbed by Disruption of pH-Mediated Left-Right Patterning

**Figure 2:**
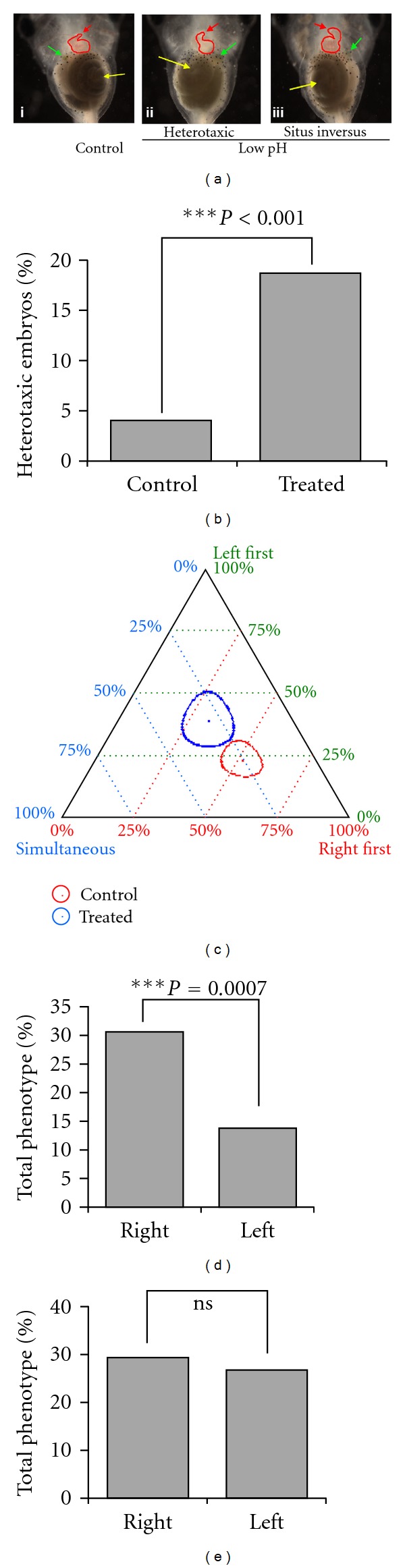
Right-ward bias in eye development is linked to the body left-right axis. (a) (i) Brightfield images of tadpoles at stage 45 showing normal positioning of the organs (*situs solitus*); rightward looping heart (red arrow), leftward coiling of gut (yellow arrow), and right side placement of gallbladder (green arrow) along the left-right axis in untreated controls. (ii)-(iii) Brightfield images of tadpoles at stage 45 after incubation in pH 4.00 0.1XMMR. (ii) Showing heterotaxic positioning of organs; rightward looping heart (red arrow) rightward coiling of gut (yellow arrow), and left side placement of gallbladder (green arrow). (iii) Showing inverse positioning of the organs (*situs inversus*); leftward looping heart (red arrow), rightward coiling of gut (yellow arrow), and left side placement of gallbladder (green arrow) along the left-right axis in tadpoles. (b) Bar graph showing percentage of embryos with heterotaxia upon incubation in 0.1XMMR (pH 4) (*n* = 481) in comparison to untreated controls (*n* = 419). The controls and treated groups were analyzed using Chi-squared test. (c) Categorical data analysis using a ternary plot shows that treatment with pH = 4 0.1XMMR that induced left-right body axis randomization also resulted in randomization and loss of the rightward bias of the polarization signal (observed via CC2-DMPE staining) involved in *Xenopus* eye development. In control embryos the polarization bias was 51% right first, 23% left first, and 26% simultaneous. The pH = 4 0.1XMMR-incubated embryos showed randomization of polarization signal 31% right first, 39% left first, and 30% simultaneous. The circles in the plot represent 95% confidence intervals. Using the calculations provided by a ternary plot algorithm (https://webscript.princeton.edu/~rburdine/stat/three_categories), the results are statistically significant (*P* < 0.05) when there is no overlap of the confidence intervals. (d) Bar graph showing right-ward bias in malformed eye upon perturbation of polarization signal. Embryos were injected with GlyR in the dorsal two cells (eye precursor cells) at the 4-cell stage and treated with IVM to induce depolarization in injected cells. Percentages of phenotypic embryos with a single malformed eye are depicted (*n* = 763). Data was analyzed using a Chi-square test comparing the right and left groups. (e) Bar graph showing no left-right bias in malformed eye upon perturbation of Pax6. Embryos were injected with DNPax6 in the dorsal two cells (eye precursor cells) at 4-cell stage. Percentages of phenotypic embryos with a single malformed eye are depicted. Data was analyzed using a Chi-squared test (*n* = 294).

We next asked whether eye asymmetry is controlled by the same pathway that governs left-right patterning of the heart and viscera. We used low-pH treatment at cleavage stages to specifically induce randomization of asymmetric gene expression and subsequent organ *situs*; this is known to interfere with the earliest steps of left-right patterning by inhibiting the proton efflux of the plasma-membrane H^+^ V-ATPase which is required for normal laterality [17]. The very early timing of this treatment ensured that it could not affect eye development directly but rather allowed us to ask whether randomization of the body's main left-right patterning pathway likewise randomized the observed physiological eye asymmetry. The efficacy of our loss-of-function treatment upon normal left-right asymmetry was confirmed by scoring the tadpoles at stage 45 for altered sidedness of the heart, gut, or gallbladder. Normal tadpoles (untreated, Figure 2(a) (i)) have a right-ward looping heart (red arrow), a left-sided gut coil (yellow arrow), and a right-sided gallbladder (green arrow). In contrast, treated tadpoles (Figure 2(a), (ii)-(iii)) showed randomization of the positions of the heart, gut, and gallbladder (independent assortment of organs), as described previously [76, 77], in the absence of generalized toxicity or other malformations (including any dorsoanterior defects). Heterotaxia was induced in 19% of embryos (Figure 2(b)) (the degree of the pH perturbation was kept well below extreme values to be compatible with continued development, to avoid dorsoanterior malformations that might confound analysis of eye development, resulting in a submaximal randomization penetrance). Each treatment (control and pH) was plotted on a ternary graph as described above; the results are considered statistically significant (*P* < 0.05, ternary plot algorithm) when there is no overlap of the 95% confidence intervals. Analysis of the polarization signal in the siblings of treated embryos at stage 18 showed that the rightward bias seen in the normal embryos (*n* = 191) (right first 51%, left first 23%, and simultaneous 26%) (Figure 2(c)) was significantly randomized in treated embryos (*n* = 105, *P* < 0.05, ternary plot algorithm) (right first 31%, left first 39%, and simultaneous 30%) (Figure 2(c)). We conclude that the determination of eye field voltage asymmetry is downstream of early ion flow dynamics, as is the positioning of the heart and viscera [8, 78]. 

### 2.3. Eye Fields Are Asymmetrically Sensitive to Perturbation of *V*_*mem*_

 To determine whether functional asymmetries (differential sensitivity to perturbation of voltage levels, as previously observed in planaria by [60]) are present in the eye field in addition to the asymmetric endogenous sequence of polarization, we experimentally depolarized the cells' *V*_mem_ using a carefully titered strategy that allowed us to manipulate *V*_mem_ and affect subtle bioelectrically controlled processes without inducing generalized toxicity or massive malformation [68]. We capitalized upon the glycine-gated chloride channel [79], which can be opened by exposure to the compound Ivermectin (IVM) [80]—a convenient method of controlling cellular potential that we have previously used to probe the role of *V*_mem_ in eye induction and metastatic-like transformation [62, 68]. Under normal conditions and standard 0.1X MMR external medium supplemented with Ivermectin, misexpression of these channels depolarizes cells (since the negatively charged chloride ion leaves GlyR-expressing cells down its concentration gradient [62]). We recently showed that injection of GlyR mRNA indeed alters the polarization pattern observed in the putative eye region and affects eye patterning [68]. 

Because the eyes are derived from the dorsal blastomeres at the 4-cell stage [88], we injected GlyR mRNA into the 2 dorsal cells and developed the embryos to stage 42. On average 45% of the affected embryos showed defects in only one eye; interestingly, there was a significant bias (2.5-fold) towards defects in the patterning of the right eye (Figure 2(d); *P* = 0.0007, Chi-squared test). As a control, we injected in a similar manner a dominant-negative *Pax6 *(DNPax6) mRNA which had been previously shown to disrupt *Xenopus *eye development [68, 89]. No statistically significant bias toward right (30%) or left (27%) eye malformation was observed among animals with only 1 eye affected (total 57% with one eye defect) (*P* > 0.05; *n* = 294 Chi-squared test in comparison with an unbiased expectation; Figure 2(e)), suggesting that the Pax6 signal during eye patterning is left-right neutral. 

From these results, we conclude that morphogenesis of the right eye is more sensitive to perturbation of *V*_mem_ than is that of the left. 

### 2.4. *K*_*ATP*_ Channel Probes Reveal Asymmetric Expression in the Eye Tissues of Developing Embryos

We next sought to determine the molecular basis for the observed asymmetry in transmembrane potential of the eye fields. Asymmetries in *V*_mem_ with significant consequences for cell behavior are often driven by differential expression of ion channels [90–92]. K_ATP_ channels [93] are octamers formed from 4 proteins from the inward-rectifying potassium family K_ir_6.x (either K_ir_6.1 or K_ir_6.2) associated with 4 sulphonylurea receptors (SUR1 and SUR2). We had previously found that altering the bioelectric state with dominant negative K_ir_ constructs that target endogenous K_ATP_ channels result in the formation of ectopic eyes [68]. Hence, we analyzed the expression pattern of K_ATP_ channel subunits in *Xenopus *embryos [94] by *in situ* hybridization at stage 18 and stage 30. Previously characterized K_ATP_ channel genes [87, 95] showed no asymmetric expression in the relevant tissues; however, probes made against the murine K_ATP_ genes revealed a striking set of expression patterns.

**Figure 3:**
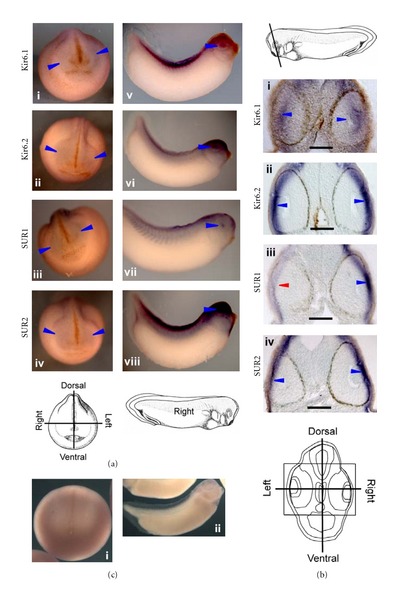
K_ATP_ channels are expressed in the putative eye regions and in the eye tissue in a left-right asymmetric manner. (a)* Xenopus* embryos at stage 18 (i)–(iv) and stage 30 (v)–(viii) were analyzed by *in situ *hybridization for K_ATP_ channel subunits showing their presence in the putative eye region ((i)–(iv) blue arrowheads) as well as in differentiated eye tissue ((v)–(viii) blue arrowheads). All four subunits (K_ir_6.1, K_ir_6.2, SUR1, and SUR2) were found to be present in the putative and developed eye tissue. In addition to the eye tissue, the K_ATP_ channel subunits were also present in the general head region and the dorsal region of the trunk. Illustration shows a stage 18 embryo with the dorsal-ventral and the left-right axes. (b) Transverse JB4 sections of *in situ *hybridized *Xenopus* embryos at stage 30 (i)–(iv) showing left-right distribution of the K_ATP_ channel subunits. Illustration shows the plane of sectioning of the stage 30 embryo. K_ir_6.1 expression is found in the inner retinal part of the eye vesicle ((i) blue arrowheads) and in the brain especially in the cells lining the ventricle and in some cells surrounding the eye tissue. K_ir_6.2, SUR1, and SUR2 expression is found in the inner layer of the 2-layer epidermis with intense staining at the lens placode ((ii)–(iv) blue arrowheads). K_ir_6.1, K_ir_6.2, and SUR2 expression is symmetric ((i), (ii), and (iv) blue arrowheads). SUR1 shows asymmetric distribution ((iii) red and blue arrowheads) where red arrowheads indicate the side with lessened expression. Scale bars = 100 *μ*m. Schematic shows a transverse section of a stage 30 embryo with the dorsal-ventral and the left-right axes indicated. (c) Sense probes showed no signal detected at neurula (i) or tailbud (ii) stages.

Probes for all four K_ATP_ channel subunits revealed signal mainly in the head, including the putative eye regions at stage 18 (Figure 3(a), (i)–(iv) blue arrowheads) and intensely stained the developing eye at stage 30 (Figure 3(a), (v)–(viii) blue arrowheads). Stage 30 embryos also showed expression in the neural tube (Figure 3(a), (v), (viii)). Stage 30 embryos were sectioned as illustrated in Figure 3(b). Probes to K_ir_6.2, SUR1, and SUR2 showed signal in the inner layer of the epidermis with intense expression in the lens placode (Figure 3(b), (ii)–(iv) blue arrowheads). K_ir_6.1 expression was found in the retinal layer of the developing eye (Figure 3(b), (i) blue arrowheads). K_ir_6.1 was also found in the cells surrounding the eye tissue and along the brain ventricle (Figure 3(b), (i)). Strikingly, expression of the SUR1 transcript was asymmetric, being expressed strongly in the right eye (Figure 3(b), (iii)-(iv), *n* = 8). No-probe controls and SUR1 and 2 sense probes, exposed for the same length of time as embryos probed with antisense RNA, exhibited no signal at any stage tested (Figure 3(c), (i), (ii)). These data reveal a novel left-right asymmetric marker distinguishing the left and right eyes at the transcriptional level. In our previous study we implicated the K_ATP_ channel in regulating the eye-specific polarization signal [68]. Although the native *Xenopus *transcripts matching the expression of these probes remain to be identified within the incompletely sequenced *X. laevis* genome, the current findings of asymmetry revealed by mouse K_ATP_ channel probes are consistent with endogenous K_ATP_ channels as a basis for the physiological differences between the left and right eyes. 

### 2.5. Melanocyte Colonization of Lateral Trunk Is Consistently Left-Right Asymmetric

**Figure 4:**
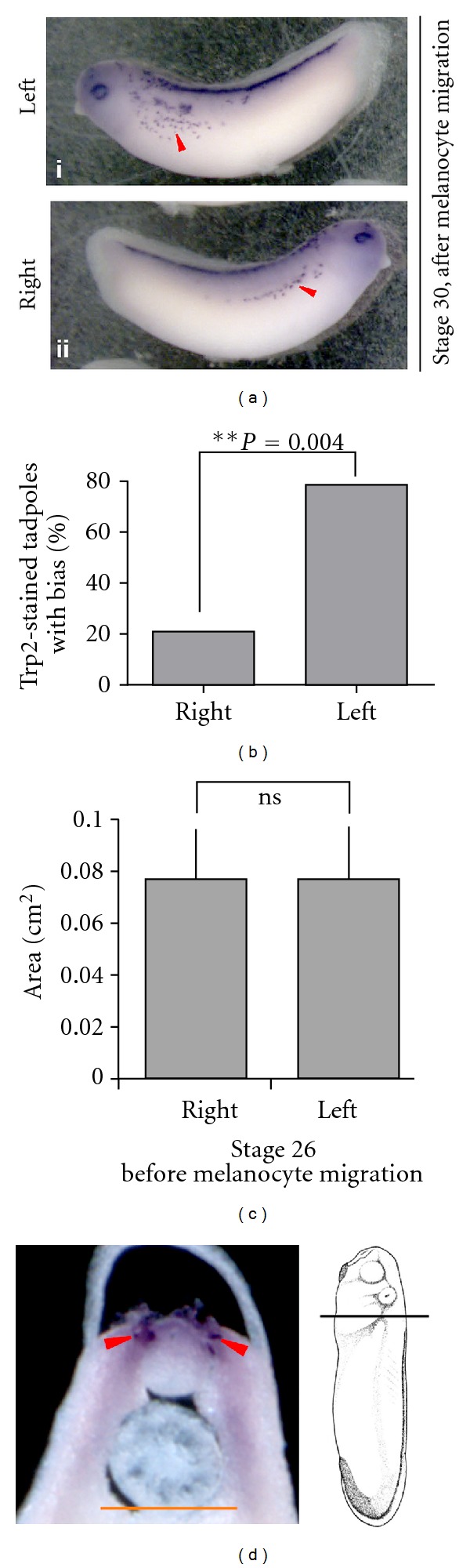
Biased migration of melanocytes along the left-right axis. (a) *Xenopus* embryos at stage 30 analyzed by *in situ* hybridization for melanocyte marker trp2 show a higher number of melanocytes on the left side (i) as compared to the right side (ii) of the embryo. Red arrowheads indicate the melanocytes being counted. (b) Quantification of a number of stage 30 embryos showing biased trp2 spots indicates that 78.6% of embryos show a leftward bias of trp2 staining. 21.4% of embryos showed higher trp2 staining on the right side. The data were analyzed using a two-tailed Binomial calculation; *n* = 28. (See Table 1 for details). (c) Quantification of *Xenopus *embryos at stage 26 analyzed by *in situ* hybridization for trp2 shows no asymmetry in melanocyte number prior to their migration. Due to dense staining at this early stage individual stained cells could not be resolved and counted, hence the stained region was marked, and the area was quantified. Quantification of the *trp2* stain on the left and right sides of embryos (*n* = 10) shows no significant difference in the staining. The areas of the signal on right and left sides were compared using a *t*-test. (d) Transverse agarose sections of *in situ* hybridized *Xenopus* embryo at stage 26 showing left-right distribution of the melanocyte marker *trp2* as measured. Illustration shows the plane of sectioning of the stage 26 embryo. Symmetric *trp2* expression (red arrowheads) is found in the area around the neural tube. Scale bar = 200 *μ*.

 The discovery of a cryptic functional and physiological asymmetry in a paired organ led us to examine other processes for asymmetries that may have heretofore escaped notice; we were especially interested in other descendants of neural precursors. Pigment cells (melanocytes, derived from neural crest cells) were identified using *in situ* hybridization with a probe against *Trp2* (also known as *Dct*), a definitive marker of melanocytes [96] at stage 30. We observed that 79% of embryos had significantly more *Trp2*-positive cells (melanocytes) on their left side compared to their right side (Figures 4(a) and 4(b) and Table 1 (*n* = 28 and *P* = 0.002, paired *t*-test)). 

**Figure 5:**
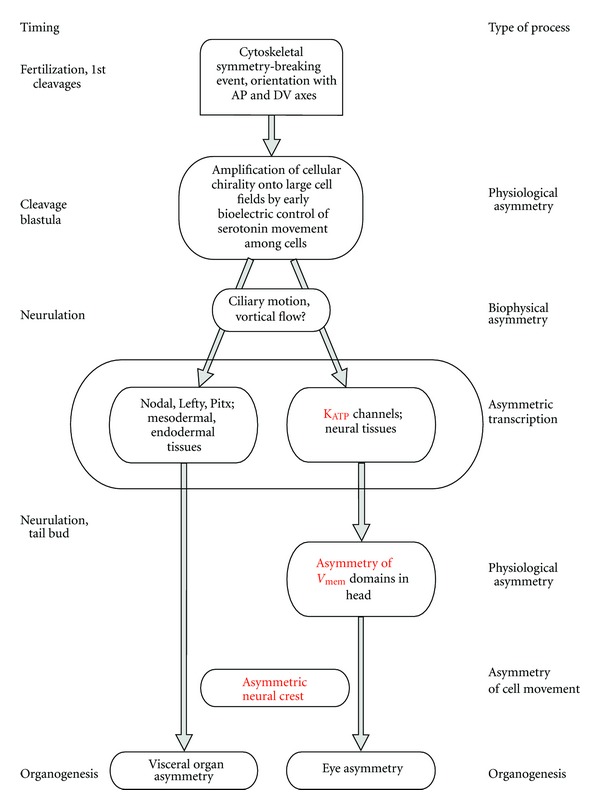
A model of physiological asymmetries within the overall scheme of the left-right patterning pathway. In *Xenopus*, bilateral symmetry is first broken, and the left-right axis is consistently oriented with respect to the dorsoventral and anterior-posterior axes, during early cleavage stages [8, 81]. The intracellular chirality is amplified onto multicellular cell fields during cleavage and blastula stages by the voltage-dependent movement of small molecule determinants through gap junctions [82–84]. The transduction of voltage gradient differences by epigenetic mechanisms [85] and other biophysical events such as ciliary movement [86] initiates at least two asymmetric transcriptional cascades. The first is the well-known Nodal, Lefty, and Pitx2 cassette that drives asymmetric organogenesis of the visceral organs. The other is the asymmetry of K_ATP_ channel subunits expressed in neural tissues, which results in asymmetric gradients of resting potential that directs development of the eye [87]. Future work will determine the functional linkage of the reported asymmetry in neural crest cell movement.

This bias in melanocyte numbers could be a result of left-right-biased differentiation of neural crest cells into melanocytes or due to biased migration of differentiated melanocytes. To test whether the observed asymmetry in melanocytes was due to asymmetric melanocyte differentiation before migration (greater number of melanocytes being produced on one side at the very beginning of melanocyte specification), we performed *in situ* hybridization with *Trp2* at an earlier stage 26 (before melanocyte migration begins). At this stage, in sections taken through the trunk, quantitative analysis of the area of *Trp2*-positive regions showed no significant difference between the right and left sides (*n* = 10) (Figure 4(c)). These results show that the left-side bias seen in melanocyte numbers at stage 30 is likely not due to biased differentiation of melanocytes from neural crest cells and favors a mechanism based on biased migration rates of differentiated melanocytes. From these results we also conclude that subtle left-right asymmetry in symmetric structures is not unique to the eye but also extends to the behavior of migratory neural crest derivatives (Figure 5). 

## 3. Discussion

### 3.1. Development of Xenopus Eyes Is Asymmetric

Previously, we showed that a specific range of relatively hyperpolarized membrane voltage regulates *Xenopus* eye development [68, 69]. While the eyes are paired organs and have been assumed to be symmetric, here we show that the bioelectric signal that endogenously regulates their formation exhibits a distinct and consistent left-right asymmetry. Hyperpolarization of the right eye occurs first (Figure 1), and the right side is consistently more sensitive to functional perturbations (Figures 2(d) and 2(e)). This observation of a right-side-first bioelectrical signal coincides with previous documentation of a morphological right-sided asymmetry of neural structures in *Xenopus*, including retina, olfactory placode, and ganglia of nerve viii [97] and is consistent with developmental asymmetries of the visual system described in flatfish [98], *Ciona intestinalis* [99], and chicken [100–102].

While consistent and statistically significant, the degree of this asymmetry (61%) is not as high as that of crucial visceral organs like heart and gut (99% in wild-type *Xenopus*). Other groups have suggested that developmental asymmetries observed in symmetric structures arise as a side effect from incomplete shielding of symmetrical structures from the left-right signaling pathway coincident in time and space and have demonstrated that retinoic acid signaling has a role in protecting developing symmetric structures from these left-right signals. Since blocking retinoic acid signaling does not exacerbate the level of asymmetry in the eye signal in our studies (Figure 1(d)), our data do not support the role of retinoic acid in the shielding of this asymmetry. However, it is possible that another (yet unknown) pathway exists that serves to reduce or mask an inherent asymmetry of the eye formation process, derived from the influence of body-wide left-right patterning signals. Another intriguing possibility is that this asymmetry is not a side effect but instead has been evolutionarily conserved due to functional relevance. 

Consistent asymmetry in handedness is seen in 60% of nonhuman primates but in 90% of humans [6, 103]. Moreover, a number of studies have reported left-right asymmetries in visual function related to food/prey and predatory responses in fish, amphibians, reptiles, birds, and mammals [104–110]. It is possible that subtle differences in the bioelectrical patterning signals of the visual system, along with asymmetries of brain and cognitive processing, are involved in establishing these behavioral and functional asymmetries. In *C. elegans,* for example, a stochastic lateral inhibition system involving ion channels results in lateralized neural differentiation and function [111–115]. It is tempting to speculate that this asymmetric bioelectric signal could trigger differential genetic, chemical, and/or biophysical patterns that overlay upon the universal eye development pattern allowing the left and right sides of the visual system to develop differing visual functionality. This is supported by our observation that depolarization-mediated disruption of endogenous bilateral polarization cues results in a right-biased disruption of eye formation. However, further functional studies will be required to test this hypothesis. In addition, this mechanism appears to be conserved, as a similar right-biased disruption of eye regeneration is observed in planaria upon depolarization [60]. Thus, comparative studies of possible asymmetries of developmental physiology and subsequent function of eyes (and other structures) among taxa may pay off in the discovery of novel asymmetries in paired organs. 

### 3.2. Eye Asymmetry versus Body Asymmetry

In the past few years, the zebrafish has become a good model for studies of asymmetry in developmental physiology, as they not only show lateralized functions but also show distinct left-right asymmetries in brain structure [116–119]. In *Xenopus*, however, no consistent marker of brain laterality has been described. The endogenous hyperpolarization described above is a convenient novel readout of neural asymmetry because it can be imaged in live animals and is thus compatible with behavioral testing and other experimental paradigms requiring the raising of animals with known neural laterality to older stages.

What is the link between the subtle asymmetries of neural derived organs and the main pathway that determines left-right asymmetry of the body? One of the fascinating aspects of left-right asymmetry determination is the disconnect between the sidedness of the major body organs and the brain. Human *situs inversus* patients (who exhibit complete reversal of the left-right body axis) show normal levels of right handedness and language lateralization [120, 121]. However, certain other behavioral traits (e.g., the hand on which the wristwatch is worn) are found to be reversed [7]. 

 In zebrafish, the asymmetry of the diencephalic region, habenula and parapineal nuclei [116] were found to be reversed in *situs inversus* animals, along with a subset of their visual laterality behavior [110]. Moreover, it is now known that individual neurites have a consistent clockwise chirality of outgrowth [122] and turning [123]. Our data in *Xenopus* demonstrate the linkage of basic body asymmetry to the cryptic asymmetry of the eye: a treatment that randomized the major visceral organs also eliminated the right-side-first bias of the eye patterning signal, suggesting that the asymmetry of the eye derives from the same pathway that patterns the major left-right axis in *Xenopus*.

### 3.3. The Physiological Origin of the Asymmetry

What is responsible for the consistently different *V*_mem_ in the left and right sides of the developing head? The K_ATP_ channel consists of K_ir_ and SUR subunits and is responsible for setting resting potential in a number of cell types [124]. K_ATP_ channel activity depends on the resting *V*_mem_, ATP levels in the cell, the external potassium levels, and the limiting levels of each subunit. Our expression data (Figure 3, which extends and complements early immunohistochemistry data on K_ATP_ channels in the hatching gland, [125]) reveal a consistent left-right asymmetry in the levels of a transcript with homology to the mouse SUR K_ATP_ channel subunit. Comparison of the mouse SUR probe sequence to the completed *Xenopus tropicalis* genome using BLAST [126] gives matches with SUR1 (*P* = 2*E* − 10), SUR2 (*P* = 2*E* − 75), but no other target (the next best match is PGER2 at nonsignificant *P* = 0.39), which supports the high probability of the probe picking up a SUR-like mRNA in *Xenopus laevis*. While we have not yet identified the native transcript corresponding to this signal, the high specificity of the staining pattern and the stringency of the *in situ* hybridization conditions make it likely that the mouse probe of SUR is picking up a native SUR-like mRNA that has not yet been characterized within the incompletely sequenced *X. laevis* genome. 

The observed asymmetry in the presence of SUR1 subunits is likely to result in a larger number of functional K_ATP_ channels on the side with greater SUR1 expression. On a background of equal initial *V*_mem_ and external potassium levels on the left and right sides, the asymmetry in subunits available for the formation of functional K_ATP_ channels may explain the difference in the *V*_mem_ on the left and right sides. It is possible that the asymmetry in functional K_ATP_ channels is also responsible for the observed asymmetry in sensitivity to eye malformation upon perturbing the *V*_mem_. Other channels may be involved in generating the observed asymmetric polarization. Comparative expression profiling and functional testing on both sides in the eye region may reveal other candidate/s that cooperate with K_ATP_ to generate the asymmetric polarization eye patterning signal.

The K_ATP_ subunits are expressed mainly in the surface ectoderm, which forms the eye placode. Interaction between the ectodermal placode and the underlying neuroectodermal and mesodermal layers results in invagination of the optic cup and eye formation [127–130]. The K_ATP_ ion channels in the surface ectoderm, and the bioelectric cell properties they regulate, may participate in modulating these signaling interactions.

One important feature of the observed bioelectric asymmetry is its temporal properties: although the right eye signal is seen first, the eye-specific *V*_mem_ signature is ultimately seen on both sides (the left side delayed by 20–30 minutes), resulting in anatomically symmetrical and equal sized eyes. As the function of ion channels is gated by posttranslational events, physiological feedback loops implemented by voltage-sensitive channels could amplify stable differences in *V*_mem_, leading to distinct voltage gradients in cell groups expressing similar complements of ion channel proteins. Future work will profile the other major conductances present in eye cells and quantitatively characterize and model the molecular-genetic and temporal details of the circuit that functions in eye precursor cells to control resting potential. Such circuits are likely to exhibit distinct stable *V*_mem_ states, as has been shown in mouse (muscle) cells to be due to potassium inward rectifying channels [131].

### 3.4. Neural Crest Derivatives Are Also Consistently Left-Right Asymmetric

A similar asymmetry was observed in neural crest-derived pigment cells, melanocytes. Melanocytes follow specific migration paths along the side of the *Xenopus* embryo after differentiation from neural crest. Interestingly, a consistent left-right asymmetry in the number of melanocytes was found upon careful analysis. Our analysis of Trp2-positive cells prior to onset of migration (stage 26) revealed that there is no apparent asymmetry in the extent of melanocyte differentiation from the neural crest cells, suggesting that the observed asymmetry is due to asymmetric migration patterns. The source of asymmetric migratory cues or mechanisms ensuring left-right asymmetrically biased response of cells to migration cues (as has been observed in mammalian cells [132, 133]) remains to be analyzed in future work. The evolutionary or developmental role of such asymmetric melanocyte distributions is not yet known; indeed, it is possible that careful quantification of cell number or other properties in other developmental events may reveal other heretofore-unrecognized consistent asymmetries in systems that are currently considered to be symmetrical. 

### 3.5. Conclusion

The identification of subtle physiological and cell positioning asymmetries in *Xenopus* development raises a number of open questions. It is possible that consistent asymmetries in bioelectric state, cell number, and sensitivity to specific perturbations remain to be discovered in many different paired (symmetric) organs. The anatomical and genetic profiling studies carried out to date may have only scratched the surface of the developmental information embedded in various somatic tissues at the level of mRNA and protein profiles and would likely not reveal physiology differences. The identification of such asymmetries, as well as their molecular origins, and the characterization of their interactions with the major left-right axial patterning cascade are likely to reveal fascinating aspects of developmental biology with significant evolutionary implications. Moreover, a mechanistic understanding of these subtle asymmetries will provide insight into the form, function, and robustness of the nervous system and may help address the lateralized diseases of both nervous and nonnervous organs. 

## 4. Materials and Methods

### 4.1. Animal Husbandry

*Xenopus laevis *embryos were collected and fertilized *in vitro* according to standard protocols [134], in 0.1X Modified Marc's Ringers (MMR; pH 7.8). *Xenopus *embryos were housed at 14–18°C and staged according to Nieuwkoop and Faber [135]. All experiments were approved by the Tufts University Animal Research Committee in accordance with the *Guide for Care and Use of Laboratory Animals*, protocol number: M2011-70.

### 4.2. Imaging of Membrane Voltage Patterns Using CC2-DMPE

CC2-DMPE (molecular probes) stocks (stock: 1 mg/mL in DMSO) were diluted 1 : 1000 in 0.1X MMR for a final concentration of 0.2 *μ*M. Stage 15–16 embryos were soaked in dye for 1.5 h. Embryos in solution were imaged using the CC2 cube set on an Olympus BX61 microscope with an ORCA digital CCD camera (Hamamatsu) with Metamorph software. See [66, 67] for additional details. For determining the bias in eye CC2-DMPE signal, embryos were imaged at regular intervals. The side of the embryo that first showed the eye CC2-DMPE signal was noted, and the embryos were kept under observation until signal was seen on both sides. Time difference between the right and left eye CC2-DMPE signals was measured by taking images at regular intervals of 10 minutes. While CC2-DMPE used alone cannot precisely quantify *V*_mem_ levels, the strong fluorescence from this positively charged dye has been shown previously to reliably identify hyperpolarized regions as confirmed by electrophysiological impalement [65–67, 136] and to identify the same locations of hyperpolarized cells in the early face as did prior studies using ratiometric imaging [69].

### 4.3. Microinjection

Capped, synthetic mRNAs were generated using the Ambion mMessage mMachine kit, resuspended in water, and injected (2.7 nL per blastomere) into embryos in 3% Ficoll. Results of injections are reported as percentage of injected embryos showing eye phenotypes, sample size (*n*) and *P* values comparing treated groups to controls. For lineage tracing, 4-cell embryos were injected with mRNA encoding *β*-galactosidase. At stage 45, fixed embryos were stained by incubation with X-gal substrate.

### 4.4. *In Situ* Hybridization

*In situ *hybridization was performed according to standard protocols [137]. Accession numbers for the sequences used as probes were K_ir_6.1 NM_008428.4, K_ir_6.2 NM_001204411.1, SUR1 NM_011510.3, and SUR2 NM_001044720.1. *Xenopus* embryos were collected and fixed in MEMFA. Prior to *in situ* hybridization, embryos were washed in PBS + 0.1% Tween-20 and then transferred to ethanol through a 25%/50%/75% series. *In situ *probes were generated *in vitro* from linearized templates using DIG labeling mix (Invitrogen, Carlsbad, CA, USA). Chromogenic reaction times were optimized to maximize signal and minimize background. Histological sections were obtained by embedding embryos after *in situ* hybridization in JB4 according to manufacturer's instructions (Polysciences). Prior to sectioning, one corner of the block was physically marked to allow unambiguous orientation of section images with respect to the left-right axis (for sections in Figure 3(b)).

#### 4.4.1. Quantification of *In Situ* Signal

 Trp2 *in situ *hybridized embryos were embedded in 4% low melting point agarose as previously described and sectioned at 100 *μ*m [138]. The sections were imaged using a Nikon SMZ1500 microscope with a Q-imaging Retiga 2000R camera. Using NIH's ImageJ software the regions exhibiting purple signal on the left and right side of the sections were marked using the freehand selection tool, and the area of the Trp2 stain was automatically quantified. The 3rd section from the last section containing eye was selected from each of the 10 different embryos and used for quantification. 

### 4.5. Heterotaxia-Inducing Treatment

 Embryos were kept in 0.1X MMR (pH 4.0–4.05) from fertilization until stage 12-13 and then moved to 0.1x MMR (pH 7.8) for the remainder of the experiment. At stage 45, the *situs* of the internal organs was scored by visual inspection of the heart, gut, and gallbladder as previously reported [17]. Categorical data analysis of the CC2-DMPE staining following drug treatment was done using ternary plots [75].

### 4.6. Retinoic Acid Receptor Inhibitor (Ro-41-5253) Treatment

 Embryos were kept in 0.1X MMR from fertilization. At ~ stage 11 Ro-41-5253 was added into 0.1X MMR with a final concentration of 1.5 *μ*M. At stage 18, a portion of the embryos were fixed and used for *in situ* hybridization. Categorical data analysis of the CC2-DMPE staining following drug treatment was done on the remaining live embryos using ternary plots [75].

### 4.7. Statistics

 All Statistical analyses were performed using Graphpad Prism (GraphPad Software, La Jolla, CA, USA), except in case of the categorical data analyses where ternary plots algorithms were used (https://webscript.princeton.edu/~rburdine/stat/three_categories). For data pooled from various iterations, Chi-square test or two-tailed Binomial calculation was used. Nonpooled data was analyzed by *t*-test (for 2 groups) or ANOVA (for more than two groups). 

## Figures and Tables

**Table 1:** Biased migration of melanocytes. Embryos were stained with the melanocyte marker Trp2 using *in situ* hybridization to identify pigment cells. The pigment spots on both the left and the right side of each embryo were counted. Student's *t*-test analysis of the raw data shows a significantly higher (*P* = 0.002) melanocyte migration on the left side of the embryos.

Embryo	Left	Right
1	25	16
2	10	9
3	18	27
4	11	13
5	7	4
6	15	17
7	29	24
8	19	13
9	15	10
10	1	2
11	27	18
12	10	6
13	8	3
14	7	3
15	45	25
16	7	4
17	11	10
18	9	14
19	7	5
20	10	4
21	6	4
22	12	7
23	6	7
24	16	8
25	15	5
26	7	5
27	10	7
28	7	4

Average	13.21	9.78

Stddev	9.11	7.08

*t*-test (paired)	0.002088	

Embryos with more melanocytes	22 (78%)	6 (22%)
